# Evolutionary mechanisms of runaway chromosome number change in *Agrodiaetus* butterflies

**DOI:** 10.1038/s41598-017-08525-6

**Published:** 2017-08-15

**Authors:** Alisa O. Vershinina, Vladimir A. Lukhtanov

**Affiliations:** 1 Department of Karyosystematics, Zoological Institute of Russian Academy of Sciences, Universitetskaya nab. 1, 199034 St. Petersburg, Russia; 2 Department of Ecology & Evolutionary Biology, University of California Santa Cruz, 95064 Santa Cruz, CA USA; 3 Department of Entomology, St Petersburg State University, Universitetskaya nab. 7/9, 199034 St. Petersburg, Russia

## Abstract

Despite predictions of the classic, hybrid-sterility model of chromosomal speciation, some organisms demonstrate high rate of karyotype evolution. This rate is especially impressive in *Agrodiaetus* butterflies that rapidly evolved the greatest chromosome number diversity known in animal kingdom within a single subgenus. Here we analyzed karyotype evolution in *Agrodiaetus* using phylogenetic comparative methods. We found that chromosome numbers possess a strong phylogenetic signal. This disproves the chromosome megaevolution model that proposes multiple chromosome rearrangements to accumulate independently in each of closely related species. We found that Brownian motion gives a more adequate description of observed trait changes than Ornstein-Uhlenbeck model. This indicates that chromosome numbers evolve via random walk along branches of the phylogeny. We discovered a correlation between karyotype changes and phylogeny branch lengths. This gradual pattern is inconsistent with the hybrid-sterility model which, due to association of major chromosome changes with cladogenetic events, predicts a high degree of punctualism in karyotype evolution. Thus, low underdominace of chromosomal rearrangements and/or prevalence of the recombination-suppression model over the hybrid-sterility model of chromosome speciation are the most common engines of the runaway chromosome number change observed.

## Introduction

Chromosomal rearrangements (CRs) play significant role in both life functioning and evolution. They are subject of studies of physiologists, geneticists and medical researchers because multiple syndromes and heritable diseases are associated with CRs^1^. CRs also attract the attention of evolutionary biologists since they trigger speciation via reducing fertility of chromosomal heterozygotes (if CRs are underdominant) or/and via suppressed recombination (if CRs are neutral and do not influence fertility of chromosomal heterozygotes)^2^. CRs maintain postzygotic isolation between well-established species and protect hybridizing lineages from merging^3^. While protecting the blocks of linked genes from recombination, CRs are crucial in adaptive evolution^4–6^. Chromosome fusions and fissions change the number of chromosomes altering the number of linkage groups^7–9^. Finally, being a part of genome architecture, chromosome rearrangements are considered to be a selectable trait *per se* ^10, 11^.

Cytological events and intracellular processes involved in chromosome evolution are relatively well-studied^12–15^, however, much less is known about evolutionary mechanisms determining probability and rate of CRs fixation in populations. Chromosome diversity in different groups of organisms show that many lineages are characterized by karyotypic constancy among species, in particular by absence or low level of interspecific variability of chromosome numbers^3, 8^. This stability is in good compliance with the fact that new chromosomal rearrangements are usually associated with heterozygote disadvantage. Therefore, their distribution and probability of fixation within a large population is low^16–18^. For example, genomes of Lepidoptera, the order which includes butterflies and moths provide an excellent example of the chromosome number stability: haploid number (n) of chromosomes n = 31 is mostly invariable and characterizes the vast majority of species, genera and families^19–22^.

In contrast to this apparent uniformity, multiple examples of chromosome number diversity within small groups of animals and plants are known. Most extreme cases of rapid chromosome evolution are found in insects, for example, in the genus *Apiomorpha* (Hemiptera: Eriococcidae) interspecific karyotypic diversity of haploid chromosome numbers from n = 2 to n = 96 was discovered^23^. Among Lepidoptera particularly striking are *Godyris* (family Nymphalidae, n = 13–120)^24^, *Leptidea* (Pieridae, n = 28–103)^7, 8, 19, 25, 26^, *Lysandra* (Lycaenidae, n = 24–93)^27^, and *Polyommatus* (Lycaenidae). In *Polyommatus* extreme variety of chromosomal numbers is concentrated within the monophyletic group of closely related *Agrodiaetus* species^3^. This West Palearctic subgenus numbers nearly 120 species, had branched roughly three million years ago and has been evolving with considerably high diversification rate^3, 28^. *Agrodiaetus* has a larger karyotype diversity than any other animal group with a total range of haploid chromosome numbers from n = 10 to n = 134^29–34^.

In vertebrates, the range of chromosome number variation between closely related species is smaller, yet still impressive: in *Corydoras* fish genus (Siluriformes: Callichthyidae)^35^ haploid numbers are ranging from n = 22 to n = 51. South American rodents of the genus *Ctenomys* (Rodentia: Ctenomiydae) have haploid chromosome numbers varying from n = 5 to n = 35 among the 60 species described^36, 37^. Muntjac deers (Artiodactyla: Cervidae) have karyotypes, ranging from n = 3 to n = 23^38^. In plants, the greatest range of within-genus karyotype variation not related to polyploidy is found in *Carex*, where haploid chromosome number ranges from n = 6 to n = 66^10, 39^.

The tempo and dynamics of such runaway chromosome number evolution are still poorly studied (for some examples see refs 3, 8, 10, 21, 39–42). Here we analyze this evolutionary phenomenon by using the butterfly subgenus *Agrodiaetus* as a model system and applying comparative phylogenetic methods in order to track ways of chromosomal changes during natural history of taxa^10, 27, 39, 42^. We were interested in the three following questions.

1. Do chromosome numbers possess a phylogenetic signal? In other words, do multiple chromosome fissions and fusions accumulate independently in each of closely related species of *Agrodiaetus*? Or, on the contrary, does this accumulation occur in rows of multiple speciation events? Phylogenetic signal has been found earlier in *Agrodiaetus* karyotype evolution by Kandul with coauthors^3^ via Abouheif’s test for serial independence. Our analysis here aims to extend their results since they did not test intensity of phylogenetic signal, neither incorporated phylogenetic uncertainty into analysis nor produced any qualitative assessment of their results.
2. Can the transformation of chromosome numbers be described by a more “neutral” model, such as Brownian motion^3^, or karyotypes evolve towards an adaptive optimum?
3. What is the temporal pattern of chromosome number change within a lifespan of species? More specifically, (3a) is there association of CR fixations with speciation events? Such association is predicted by the classic chromosome-sterility theories of chromosome speciation and stems from the idea of fixation of a single underdominant CR^17, 43^.

If CR fixation correlates with speciation, we would expect a strong punctualism in chromosome evolution with the majority of CRs associated with cladogenetic events at the very initial stages of species diversification. Alternatively, (3b) CRs changing the chromosome number do not seriously impact fertility, can accumulate gradually and are not necessarily associated with early stages of speciation.

## Results

### Phylogeny reconstruction

**Figure 1:**
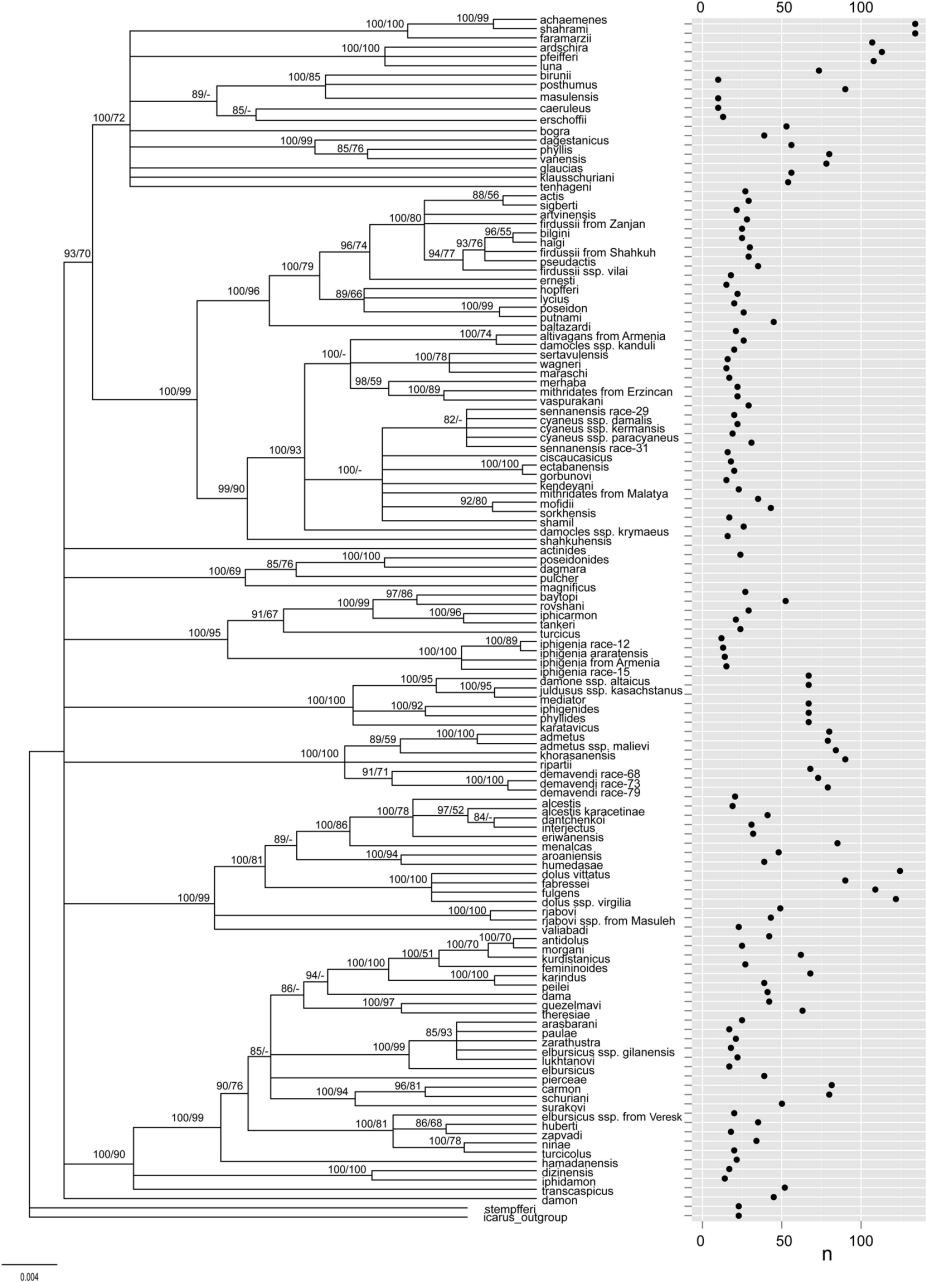
Bayesian majority rule consensus phylogram of seven nuclear and mitochondrial DNA markers for *Agrodiaetus* species. Numbers above the branches indicate Bayesian posterior probabilities and bootstrap support values for maximum likelihood tree (ML), in that order, expressed as percentages, dashes indicate support values below 50%. Dots on the plot represent haploid chromosome numbers (n).

Phylogenetic trees using Maximum Likelihood (ML) and Bayesian Inference (BI) methods were obtained for 130 populations corresponding to 111 species comprising all the sequence data available to date for 120 described *Agrodiaetus* species (see Supplementary Table S1 for the sequence IDs). BI was performed with the following models: GTR + I + Г for mitochondrial markers *COI* and *COII* and HKY85 for *leu*-*tRNA*; GTR for nuclear *5*.*8S rDNA* + *ITS2* + *28S rDNA*. ML analysis was performed with GTR model for the entire dataset (ML of the most plausible tree was −ln = 19652.9). BI consensus tree topology was mostly the same as ML tree topology. Figure 1 shows the calculated BI consensus tree. Most nodes were strongly supported; more than half of all nodes had Bayesian posterior probabilities of 0.90 and higher.

### Phylogenetic signal

**Figure 2:**
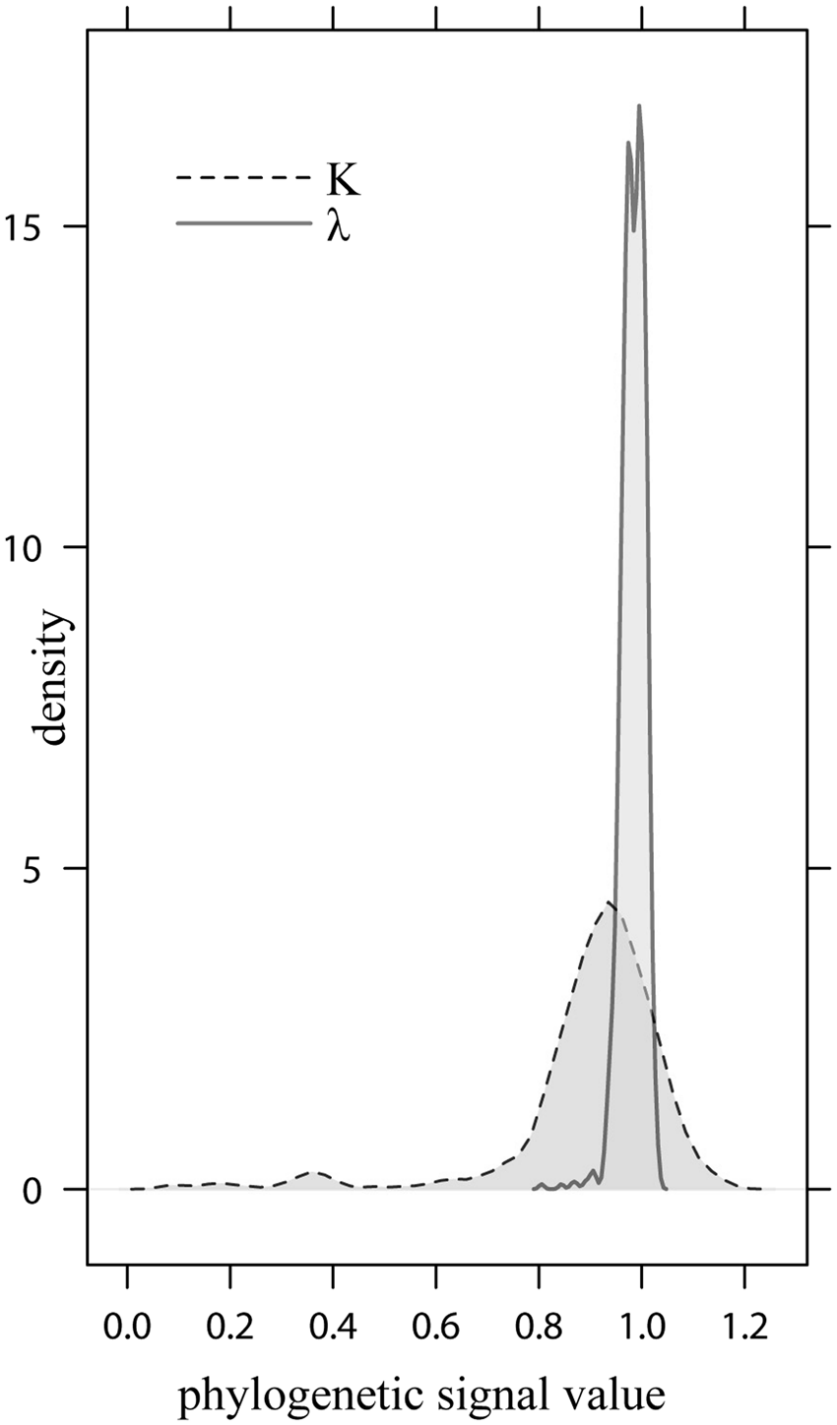
Density distribution of phylogenetic signal for Pagel’s lambda and Blomberg’s K metrics across 1000 Bayesian post burn-in phylograms for subgenus *Agrodiaetus*.

We calculated the phylogenetic signal using Pagel’s lambda and Blomberg’s K metrics^44, 45^. As shown in the Fig. 2, K and lambda vary in relation to tree topology (including different branch lengths), but for most phylograms both K and lambda approach the value indicating strong phylogenetic signal (K = 1, λ = 1). Pagel’s lambda has varied from λ = 0.81 to λ = 1.03 and Blomberg’s K from 0.06 to 1.4 (with K = 0.9 and K = 1 for the most tree topologies). Obtained K and lambda values were significantly different than expected by chance (*p* < 0.001). Lambda values were tested using likelihood ratio tests where topologies with λ = 0 and λ = 1 were compared against each other and against maximum likelihood lambda values accordingly. Maximum likelihood values of lambda were significantly greater than 0, however maximum likelihood λ value was not distinguished from the modelled λ = 1 (*p* < 0.001).

### Brownian motion versus Ornstein-Uhlenbeck model

**Figure 3:**
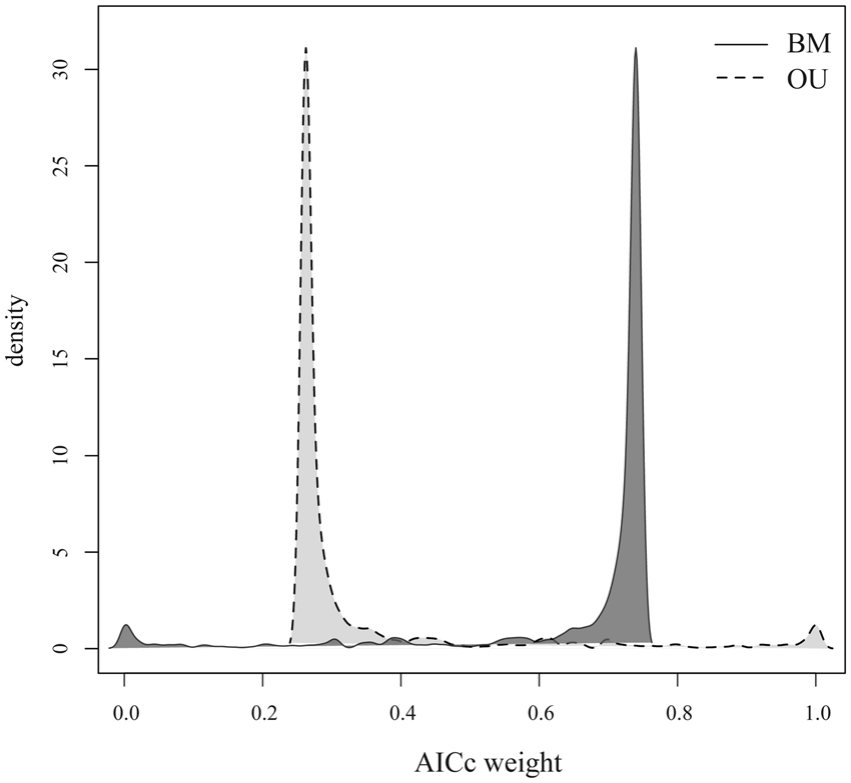
Density distribution of Brownian motion (BM) and Ornstein–Uhlenbeck (OU) models support over 1000 Bayesian post burn-in phylograms for *Agrodiaetus* species. AICc is Akaike information criterion with a correction for finite sample sizes. AICc weights are the relative likelihood of each model: the weight is smaller if the model is less plausible.

The Brownian motion (BM) as a constant variance process is often employed to model and characterize the continuous quantitative trait evolution, which is controlled by influence of stochastic factors^46–50^. Alternatively Ornstein-Uhlenbeck process (OU) is the commonly used to model selective pressure towards a particular range of phenotypes indicating an adaptive optimum^50, 51^. BM and OU processes were compared via corrected Akaike information criteria (AICc)^52^. Akaike weights demonstrated the higher likelihood rate for BM model (Fig. 3). Thus, we conclude that BM model gives a more adequate description of observed trait changes than the OU model.

### Gradual versus punctual mode of chromosome evolution

**Figure 4:**
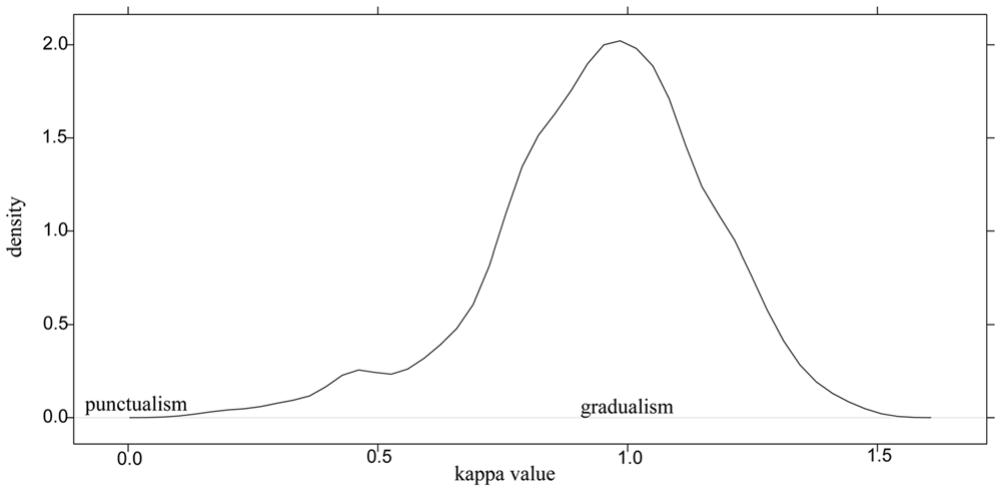
Density distribution of maximum likelihood tree-scaling parameter kappa values across 1000 Bayesian post burn-in phylograms for *Agrodiaetus* species.

**Figure 5:**
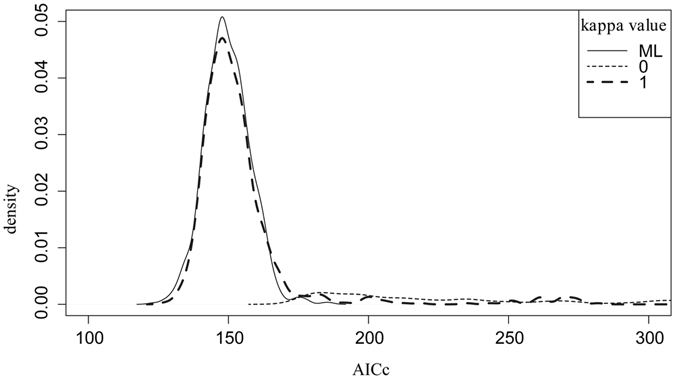
Density distribution of Pagel’s kappa model support over 1000 Bayesian post burn-in phylograms for *Agrodiaetus* species. AICc is Akaike information criterion riteria with a correction for finite sample sizes, the smaller the AICc means the more plausible the model.

To test for a punctuational versus gradual mode of trait evolution we used κ (kappa) parameter^53^. Testing kappa values using AICc demonstrated that likelihood of κ = 1 and AICc of maximum likelihood kappa coincided almost entirely, whereas AICc of other models (κ = 0 and κ = 3) were higher (AICc of κ = 3 model is approximately 20 times as high as κ = 1 AICc), suggesting that κ = 1 most likely reflects mode of karyotype evolution indicating clear gradualism in CRs (Figs 4 and 5).

## Discussion

The observed diversity of chromosome numbers in *Agrodiaetus* could theoretically be a result of multiple CRs emerged from an ancestral karyotype and independently accumulated in each of the studied species. This pattern of chromosome change has been described by Baker and Bickham^54–56^ under the name “karyotype megaevolution”. This model describes a fast accumulation of multiple CRs occurring independently in each species which results in a lack of phylogenetic signal. Visual inspection of the *Polyommatus* (*Agrodiaetus*) chromosome numbers mapped on the consensus Bayesian phylogeny (Fig. 1) shows that some sister species have extremely different chromosome numbers (e.g. n = 10 in *P*. *masulensis* and n = 85 in *P*. *posthumus*). These drastically different karyotypes are not a result of polyploidy but a consequence of independent chromosome fissions and fusions^29^. Such a case may seem, at the first glance, to correspond with the karyotype megaevolution model.

An alternative to the chromosomal megaevolution is the model of stepwise accumulation of similar CRs in consequent rows of speciation events resulting in strong phylogenetic signal^8^. As our estimates show, this is certainly the case for the chromosome number evolution in *Agrodiaetus*. The studied karyotypes are phylogenetically nonindependent: CRs such as fusions and fissions are likely to pile up in *Agrodiaetus* during a succession of multiple speciation events and incrementally result in low and high chromosome numbers. Consequently, closely related taxa tend to have similar chromosome sets. Furthermore, strong phylogenetic signal also suggests that recently diverged species are more karyotypically akin than deeply diverged ones: the expected difference in chromosome sets between the descendants grows proportional to the time since they shared a common ancestor.

The strong phylogenetic signal does not describe a particular process or rate of evolution^57, 58^. Previously BM model has been hypothesized to characterize rapid karyotype transformations in *Agrodiaetus* ^3^. The model implies gradual changes through time to occur aside from the current character state and expected mean of the changes^50, 59^. Our current study confirms that in *Agrodiaetus* dynamics of chromosome evolution follows Brownian motion and can be described as neutral drift (or “random walk”) with changes around a mean of zero.

Since karyotypes may fall into the selective optimum or equilibrium, we checked also the presence of adaptive peak in chromosome numbers by testing OU model against BM. The idea of the adaptive shift in chromosomal numbers reflects certain conceptions. First of all, quantum and stasipatric chromosomal speciation models imply adaptive chromosome re-patterning via spontaneous gene and linkage group rearrangements^17, 60^. Second, spatial constraints can also occur because chromosome size and shape are important in the segregation of chromosomes during the cell division^29^. There also may be special cases of adaptivity in chromosome organization, such as eu- and heterochromatin adaptation to nocturnal and diurnal vision in mammals^61^.

On a range of different tree topologies modelling results conclusively demonstrate that BM, but not OU fits the data (Fig. 3). Therefore, it is likely that every next step in chromosome evolution along a phylogenetic lineage is independent of previous ones with variance accumulating over time. Thereby CRs and karyotypes depend on phylogenesis but probably not on any adaptive regime or evolutionary constraint.

If underdominant CRs directly initiate speciation via formation of post-zygotic reproductive isolation, as suggested in classic theories of chromosomal evolution^17, 43^, their fixation should be associated with the points of cladogenesis resulting in strong punctual mode of karyotype evolution. If CRs are not underdominant (or at least not strongly underdominant), their fixation and accumulation is expected to be gradual without any association between CRs and with cladogenesis. Graduality is consistent with at least three different scenarios. First, CRs might be irrelevant for reproductive isolation^17^. Second, according to the recombination-suppression model^2^, CRs might promote parapatric speciation working as a genetic filter between populations^60, 62–64^. Recombination-suppression model implies that mutations associated with the rearranged parts of the genomes are protected from intrapopulation gene flow. This results in accumulation of genetic incompatibilities leading to reproductive isolation and eventual speciation. Third, cascade^43^ and monobrachial^65^ models postulate the sequential accumulation of several neutral or nearly neutral CRs in independent populations resulting in interpopulation post-zygotic isolation.

In this study, we discovered a correlation between number of CRs and branch length, and this pattern is in a good agreement with gradual accumulation of CRs in *Agrodiaetus*. AICc conclusively demonstrate that κ = 1 model fits the data better κ = 0 or κ = 3 (Fig. 5). Thus, our data are hardly compatible with the classic chromosomal hybrid sterility model being the main engine of chromosome number diversity in *Agrodiaetus*. However, the results are well consistent with the mentioned above scenarios of karyotype evolution in which chromosome changes indirectly or weakly affect fertility of heterozygotes for CRs.

In Lepidoptera, there are other empirical data suggesting that chromosome fusions and fissions are not strongly underdominant and can accumulate gradually. There is an experimental evidence that *Antherea* moths heterozygous for multiple CRs are not sterile^66^. In *Leptidea sinapis* butterflies (Lepidoptera, Pieridae) the diploid chromosome number gradually decreases from 2n = 106 in Spain to 2n = 56 in eastern Kazakhstan, resulting in a 6000 km-wide cline where within-species accumulation of chromosomal changes is shown^7, 8, 67^. Homoploid hybrid speciation in *Agrodiaetus* via hybridization between chromosomal races with n = 27 and n = 68 and consequent chromosome sorting^33^ assumes that F_1_ hybrids heterozygous for 41 fusions/fissions were fertile. Thus, it seems unlikely that CRs are strongly underdominant in these groups. Interestingly, low underdominance of chromosomal fusions and fissions is not restricted to Lepidoptera. For example, it is also known in mammals^68–70^.

Finally, we should note that Lepidoptera, including *Agrodiaetus* butterflies, have holocentric chromosomes lacking localized centromeres and distinct chromosome arms^71–73^. Due to this special organisation fissions and fusions do not dramatically alter meiotic segregation as in the case of monocentric chromosomes which may became acentric after fission or dicentric after fusion. Higher viability of chromosomes after fragmentations and fusions and other phenomena related to holocentric structure such as holokinetic drive^74^ also could contribute to the origin of chromosomal diversity. Nevertheless, we believe that low underdominace of chromosomal rearrangements and prevalence of recombination-suppression over hybrid-sterility model are most common engines of runaway evolution of chromosome numbers in Lepidoptera.

## Methods

### Phylogeny reconstruction

Sequences (5.8S rDNA partial gene, *ITS2* complete and 28S rDNA partial, *COI*, *leu*-*tRNA* complete and *COII* – partial) were collected from GenBank. Sequences of each gene were aligned separately by clustalW, the alignments were corrected manually using BioEdit^75^. Since *ITS2* sequence has multiple indels which are highly specific on the species-level, it provides additional information for phylogenetic analysis^76, 77^, so we treated all *ITS2* indels as binary characters (insertion - 1, deletion - 0). The final concatenated alignment had length of 2948 nucleotides (*COI* 1–1539 bp, *leu*-*tRNA* 1540–1604 bp, *COII* 1605–2281 bp, *5*.*8 S rDNA* + *ITS2 *+* 28 S rDNA* 2282–2948 bp) and 23 binary characters. Additional 643 bp fragment of *COI* and 592 bp fragment of *ITS2* for *P*. *kendevani* (*ITS2*), *P*. *luna* (*ITS2*), and *P*. *shahukuhensis* (*ITS2*) were kindly provided by Nazar Shapoval, Zoological Institute, RAS.

As an outgroup, we used *P*. *icarus* (Rottemburg, 1775) and *P*. *stempfferi* (Brandt, 1938) since they were earlier inferred as the outgroups to the subgenus *Agrodiaetus* ^28^. Substitution models were inferred by the hierarchical likelihood ratio, Akaike and Bayesian information criteria tests, as implemented in jModelTest2^78^, models were evaluated separately for each gene. ML analysis was conducted using RAxML Black Box^79^ on the CIPRES Science Gateway platform^80^. Ten independent search replicates were run under the GTR model. Bootstrap support values for nodes on the ML topology were computed with RAxML rapid bootstraping algorithm^81^ by running 700 bootstrap replicates. Bayesian analysis was conducted using MrBayes 3.2^82^ on four molecular (*COI*, *COII*, *leu*-*tRNA* and *5*.*8S rDNA* + *ITS2* + *28S rDNA* genes) and one “standard” (binary) partitions using 7 million generations. Two independent runs were performed, each with four chains (three heated and one cold), using independent-gamma rate relaxed clock model with uniform branch lengths. Trees were sampled at intervals of every 2,000 generations. Stationarity was determined by examining log-likelihood scores plotted across generations with Tracer (http://tree.bio.ed.ac.uk/software/tracer/), plotting posterior probabilities of all splits for paired MCMC runs by AWTY on-line service^83^ and by examining standard deviation of split frequencies between the two runs for convergence. Of the 3,501 trees sampled in each run, first 5% were discarded as burn-in and the remaining trees were used to construct a 50% majority rule consensus phylogram.

### Phylogenetic signal and mode of evolution

Following approach suggested by Hipp^10^ and taking into account the lack of visual markers in holocentric chromosomes, we used only chromosome number “as a proxy for karyotype” and modeled chromosome number evolution as an evolution of a quantitative continuous character. Frequency of chromosome fusions and fissions depends on the number of chromosomes. Therefore, haploid chromosome numbers were log-transformed prior to phylogenetic comparative analysis.

To make the sampling as complete as possible we used all chromosome data available, including our own previously published results^3, 30–34, 76, 84–91^. In the analysis, the majority of the tips on the phylogenetic tree refer to species (Supplementary Table S1). In cases of intraspecific chromosomal variations, chromosomal races (i.e., populations with stable differentiated karyotypes) were used as entities for phylogenetic comparative analysis (in this cases tips on the phylograms refer to populations). Such cases were found in *P*. *cyaneus*, *P*. *damocles*, *P*. *demavendi*, *P*. *dolus*, *P*. *elbursicus*, *P*. *firdussii*, *P*. *iphigenia*, *P*. *mithridates*, *P*. *rjabovi* and *P*. *sennanensis*. For five species karyotype data are absent and we excluded them from the phylogenetic comparative analysis (*P*. *actinides*, *P*. *dagmara*, *P*. *magnificu*s, *P*. *mediator*, *P*. *pulcher*). To incorporate phylogenetic uncertainty into analysis the entire comparative approach was carried out on 1000 ultrametric randomly chosen post burn-in phylograms^92^.

All computational procedures were performed using R packages for phylogenetic comparative methods: ape^93^, geiger: fitContinuous^94^, caper: pgls (https://r-forge.r-project.org/projects/caper/), phytools: phylosig^95^ and qpcR: akaike.weights (https://r-forge.r-project.org/projects/qpcr/).

## Data Availability

All data analyzed during this study are included in this published article and its Supplementary Information file.

## Electronic supplementary material

Supplementary Table S1
