# Supplementary material for: Evolutionary mechanisms of runaway chromosome number change in Agrodiaetus butterflies
Source: Sci Rep. 2017 Aug 15;7:8199. doi: 10.1038/s41598-017-08525-6 (PMC5557896; doi:10.1038/s41598-017-08525-6)
Supplement: Supplementary file 1 — Supplementary Table S1 [file 41598_2017_8525_MOESM1_ESM.doc]

Evolutionary mechanisms of runaway chromosome number change in *Agrodiaetus* butterflies

Alisa O. Vershinina and Vladimir A. Lukhtanov

**Supplementary information**

## Supplementary Table S1. Karyotypes and *COI*, *trnL*, *COII*, *5.8S rDNA*, *ITS2*, *28S rDNA* sequence data for the studied samples of the subgenus *Agrodiaetus*.

| *Modal chromosome number (in brackets) reflects haploid karyotype in most of the individuals studied. "NSh" abbreviation corresponds to the Nazar Shapoval's sequence ID. | | | | |
| --- | --- | --- | --- | --- |
| **species name** | **n (modal n)*** | **Sequence ID** | | |
|  |  | **COI+trnL+COII** | **COI** | **5.8S rDNA + ITS2+28S rDNA** |
| icarus (outgroup) | 23 | AY496815 | AY556927 | AY556590 |
| achaemenes | 134 | EF104615 | AY557140 | AY556740 |
| actinides | NA | EF104621 | GU559748 | AY556753 |
| actis | 26-28 (27) | EF104606 | AY556999 | AY556633 |
| admetus | 80 | AY496711 | AY556867 | AY556733 |
| admetus ssp. malievi | 79 | EF104617 | - | HM210176 |
| alcestis | 20-21 (20) | - | AY557008 | AY556641 |
| alcestis ssp. karacetinae | 19 | AY954018 | - | AY556574 |
| altivagans | 20- 23 (21) | AY496716 | AY556846 | AY556717 |
| antidolus | 42-44 (42) | AY496717 | AY557095 | AY556692 |
| zarathustra | 20-22 (21) | AY953994 | AY557141 | AY556741 |
| arasbarani | 25 | AY496718 | - | - |
| ardschira | 113 | AY954001 | - | - |
| aroaniensis | 48 | - | AY556725 | AY556856 |
| artvinensis | 21-22 (21) | - | AY557038 | AY556663 |
| baltazardi | 45 | AY954008 | - | - |
| baytopi | 27-28 (27) | AY496720 | AY557087 | AY556688 |
| bilgini | 25 | AY496721 | - | - |
| birunii | 10 | AY953985 | AY556912 | AY556578 |
| bogra | 53 |  | NSh J318 |  |
| caeruleus | 10 | AY953987 | AY556926 | AY556589 |
| carmon | 81-82 | AY496722 | AY556981 | AY556622 |
| ciscaucasicus | 16 | AY496724 | - | - |
| cyaneus ssp. damalis | 20 | EF104610 | AY557143 | - |
| cyaneus ssp. kernmansis | 22 | AY954003 | - | - |
| cyaneus ssp. paracyaneus | 19 | AY953993 | - | - |
| dagestanicus | 39-40 (39) | AY954025 | - | - |
| dagmara | NA | EF104618 | AY556852 | - |
| dama | 41 | - | AY557007 | AY556640 |
| damocles ssp. kanduli | 25 | AY496726 | - | - |
| damocles ssp. krymaeus | 26 | AY496727 | - | HM210178 |
| damon | 45 | AY496732 | AY557131 | AY556714 |
| damone ssp. altaicus | 67 | AY496734 | FJ663228 | - |
| dantchenkoi | 40-42 | AY496737 | AY557073 | AY556679 |
| demavendi race-68 ssp. lorestanus | ca68 | AY953995 | AY557142 | AY556743 |
| demavendi race-73 | 73 | EF104630 | - | - |
| demavendi race-79 | 79 | AY954007 | - | - |
| dizinensis | 17 | EF104638 | AY556939 | AY556599 |
| dolus ssp. virgilia | 122 | HM210162 | - | HM210180 |
| dolus ssp. vittatus | 124-125 (124) | AY496740 | - | - |
| ectabanensis | 18 | AY953992 | - | - |
| elbursicus | 17 | AY953999 | AY556877 | AY556555 |
| elbursicus from Veresk | 20 | AY953986 | - | - |
| elbursicus ssp. gilanensis | 18 | EF104637 | - | - |
| eriwanensis | 29-35 (32) | AY496742 | - | - |
| ernesti | 18 | - | AY556989 | AY556626 |
| erschoffii | 13-15 (13) | AY496743 | AY556925 | AY556588 |
| fabressei | 90 | AY496744 | AY556952 | AY556608 |
| faramarzii | 107 | AY954017 | - | - |
| femininoides | 27 | EF104636 | AY557150 | AY556749 |
| firdussii from Shahkuh | 30-34 (30) | AY953997 | - | - |
| firdussii ssp. vilai | 35 | EF104607 | - | - |
| firdussii from Zanjan | 28 | EF104623 | - | AY556576 |
| fulgens | 108-110 (109) | AY496712 | AY556954 | AY556610 |
| glaucias | 56 | AY496747 | AY557134 | AY556736 |
| gorbunovi | 20 | AY954022 | AY556899 | AY556569 |
| guezelmavi | 41-42 (42) | - | AY557022 | AY556651 |
| haigi | 25 | AY496750 | AY557069 | AY556677 |
| hamadanensis | 21-22 | AY502112 | AY556875 | AY556554 |
| hopfferi | 15 | AY496751 | AY557005 | AY556638 |
| huberti | 35-37 (35) | AY496753 | AY557123 | AY556707 |
| humedasae | 39 | HM210170 | AY557127 | HM210193 |
| interjectus | 29-32 (31) | - | AY557059 | AY556671 |
| iphicarmon | 29 | EF104608 | AY556990 | AY556627 |
| iphidamon | 14 | AY953988 | AY556913 | AY556584 |
| iphigenia race-12 | 12 | AY496757 | AY557061 | AY556656 |
| iphigenia from Armenia | 14 | AY556849 | - | - |
| iphigenia ssp. araratensis | 13 | AY496756 | - | - |
| iphigenia race-15 | 15 | EF104609 | - | - |
| iphigenides | 65-67 (67) | AY496758 | FJ663236 | AY556722 |
| juldusus ssp. kasachstanus | 67 | AY496759 | - | - |
| karatavicus | 67 | AY496760 | - | - |
| karindus | 68-73 (68) | EF104633 | AY557145 | NSh E398 |
| kendevani | 15-17 (15) | AY954005 | - | NSh Nz3 |
| khorasanensis | 84 | AY954013 | AY557138 | AY556737 |
| klausschuriani | 56 | AY953984 | AY556910 | AY556577 |
| kurdistanicus | 62 | AY496762 | AY557074 | AY556680 |
| lukhtanovi | 22 | AY954021 | - | - |
| luna | 73-74 | EF104624 | - | NSh E154 |
| lycius | 21-22 (22) | - | AY556985 | AY556625 |
| magnificus | NA | EF104619 | - | - |
| marasсhi | 16 | - | AY557000 | AY556634 |
| masulensis | 10 | EF104613 | - | GQ166175 |
| mediator | NA | EF104602 | - | - |
| menalcas | 85 | AY496763 | AY557001 | AY556635 |
| merhaba | 16-17 (17) | AY496764 | AY557037 | AY556662 |
| mithridates from Etzincan | 21-27 (22) | AY496765 | - | - |
| mithridates from Malatya | 21-27 (23) | - | AY557006 | AY556639 |
| mofidii | 35 | AY954012 | AY557137 | AY556739 |
| morgani | 25-27 (25) | - | NSh Z524 | NSh W154 |
| ninae | 33-35 (34) | AY496766 | AY557113 | AY556701 |
| paulae | 17 | - | AY556892 | AY556564 |
| peilei | 38-39 (39) | EF104634 | AY557144 | AY556744 |
| pfeifferi | 106-108 (108) | AY954002 | - | - |
| phyllides | 66-67 (67) | AY496770 | FJ663239 | - |
| phyllis | 80 | AY953989 | AY556923 | AY556587 |
| pierceae | 22 | AY496773 | AY557083 | AY556686 |
| poseidon | 19-21 (20) | AY496775 | AY557002 | AY556636 |
| poseidonides | 24 | EF104622 | - | AY556721 |
| posthumus | 90 | AY953990 | AY556922 | AY556586 |
| pseudactis | 29 | AY496777 | AY556845 | AY556716 |
| pulcher | NA | EF104620 | - | - |
| putnami | 26 | AY496778 | AY557112 | AY556700 |
| ripartii | 90 | EF104603 | AY556962 | AY556727 |
| rjabovi | 49 | AY954019 | - | - |
| rjabovi from Masuleh | 43 | AY954006 | - | - |
| rovshani | 52-53 | AY496788 | AY556897 | AY556567 |
| sennanensis race-29 | 29 | EF104616 | - | - |
| sennanensis race-31 | 31 | EF104625 | AY557147 | AY556746 |
| sertavulensis | 20 | - | AY557023 | AY556652 |
| shahkuhensis | 16 | AY953998 | - | NSh Nz7 |
| shahrami | 134 | AY954016 | AY557154 | AY556752 |
| shamil | 17 | AY954024 | - | - |
| sigberti | 29 | - | AY557020 | AY556650 |
| sorkhensis | 43 | AY954010 | - | - |
| stempfferi | 23 | AY954000 | - | JX093393 |
| surakovi | 50 | AY496792 | AY556844 | AY556715 |
| tankeri | 20-21 (21) | AY496794 | AY557125 | AY556709 |
| tenhageni | 54 | AY954009 | AY557139 | AY556738 |
| theresiae | 63 | - | AY557013 | AY556645 |
| transcaspicus | 52-53 (52) | AY954014 | - | - |
| turcicolus | 20 | AY496796 | AY557110 | AY556699 |
| turcicus | 24 | AY496798 | AY557117 | AY556674 |
| valiabadi | 23 | - | AY556934 | AY556594 |
| vanensis | 78 | AY496799 | AY556850 | AY556720 |
| vaspurakani | 22 | AY496713 | AY557085 | AY556687 |
| wagneri | 16-17 (16) | - | AY556995 | AY556629 |
| zapvadi | 18-19 (18) | AY496741 | AY557067 | AY556675 |
| schuriani | 75-80, 81-82 (80) | - | AY557014 | AY556646 |
